# Challenges and solutions to system-wide use of precision oncology as the standard of care paradigm

**DOI:** 10.1017/pcm.2024.1

**Published:** 2024-03-26

**Authors:** Nesrine Lajmi, Sofia Alves-Vasconcelos, Apostolos Tsiachristas, Andrew Haworth, Kerrie Woods, Charles Crichton, Theresa Noble, Hizni Salih, Kinga A. Várnai, Harriet Branford-White, Liam Orrell, Andrew Osman, Kevin M. Bradley, Lara Bonney, Daniel R. McGowan, Jim Davies, Matthew S. Prime, Andrew Bassim Hassan

**Affiliations:** 1 Diagnostics Division, Roche Information Solutions, F. Hoffmann-La Roche Ltd., Basel, Switzerland; 2 Oxford Molecular Pathology Institute, Sir William Dunn School of Pathology, University of Oxford, Oxford, UK; 3 Nuffield Department of Primary Care Health Sciences, Radcliffe Observatory Quarter, Oxford, UK; 4 Oxford University Hospitals NHS Foundation Trust, Oxford, UK; 5 Roche Healthcare Consulting, Roche Diagnostics Limited, West Sussex, UK; 6 Wales Research and Diagnostic PET Imaging Centre, University Hospital of Wales, Cardiff, UK; 7 Big Data Institute, Li Ka Shing Centre for Health Information and Discovery, Oxford, UK

**Keywords:** precision medicine, precision oncology, cost-effectiveness, health data, patient record, interoperability, standard of care, patient centred

## Abstract

The personalised oncology paradigm remains challenging to deliver despite technological advances in genomics-based identification of actionable variants combined with the increasing focus of drug development on these specific targets. To ensure we continue to build concerted momentum to improve outcomes across all cancer types, financial, technological and operational barriers need to be addressed. For example, complete integration and certification of the ‘molecular tumour board’ into ‘standard of care’ ensures a unified clinical decision pathway that both counteracts fragmentation and is the cornerstone of evidence-based delivery inside and outside of a research setting. Generally, integrated delivery has been restricted to specific (common) cancer types either within major cancer centres or small regional networks. Here, we focus on solutions in real-world integration of genomics, pathology, surgery, oncological treatments, data from clinical source systems and analysis of whole-body imaging as digital data that can facilitate cost-effectiveness analysis, clinical trial recruitment, and outcome assessment. This urgent imperative for cancer also extends across the early diagnosis and adjuvant treatment interventions, individualised cancer vaccines, immune cell therapies, personalised synthetic lethal therapeutics and cancer screening and prevention. Oncology care systems worldwide require proactive step-changes in solutions that include inter-operative digital working that can solve patient centred challenges to ensure inclusive, quality, sustainable, fair and cost-effective adoption and efficient delivery. Here we highlight workforce, technical, clinical, regulatory and economic challenges that prevent the implementation of precision oncology at scale, and offer a systematic roadmap of integrated solutions for standard of care based on minimal essential digital tools. These include unified decision support tools, quality control, data flows within an ethical and legal data framework, training and certification, monitoring and feedback. Bridging the technical, operational, regulatory and economic gaps demands the joint actions from public and industry stakeholders across national and global boundaries.

## Graphical abstract

**Figure:**
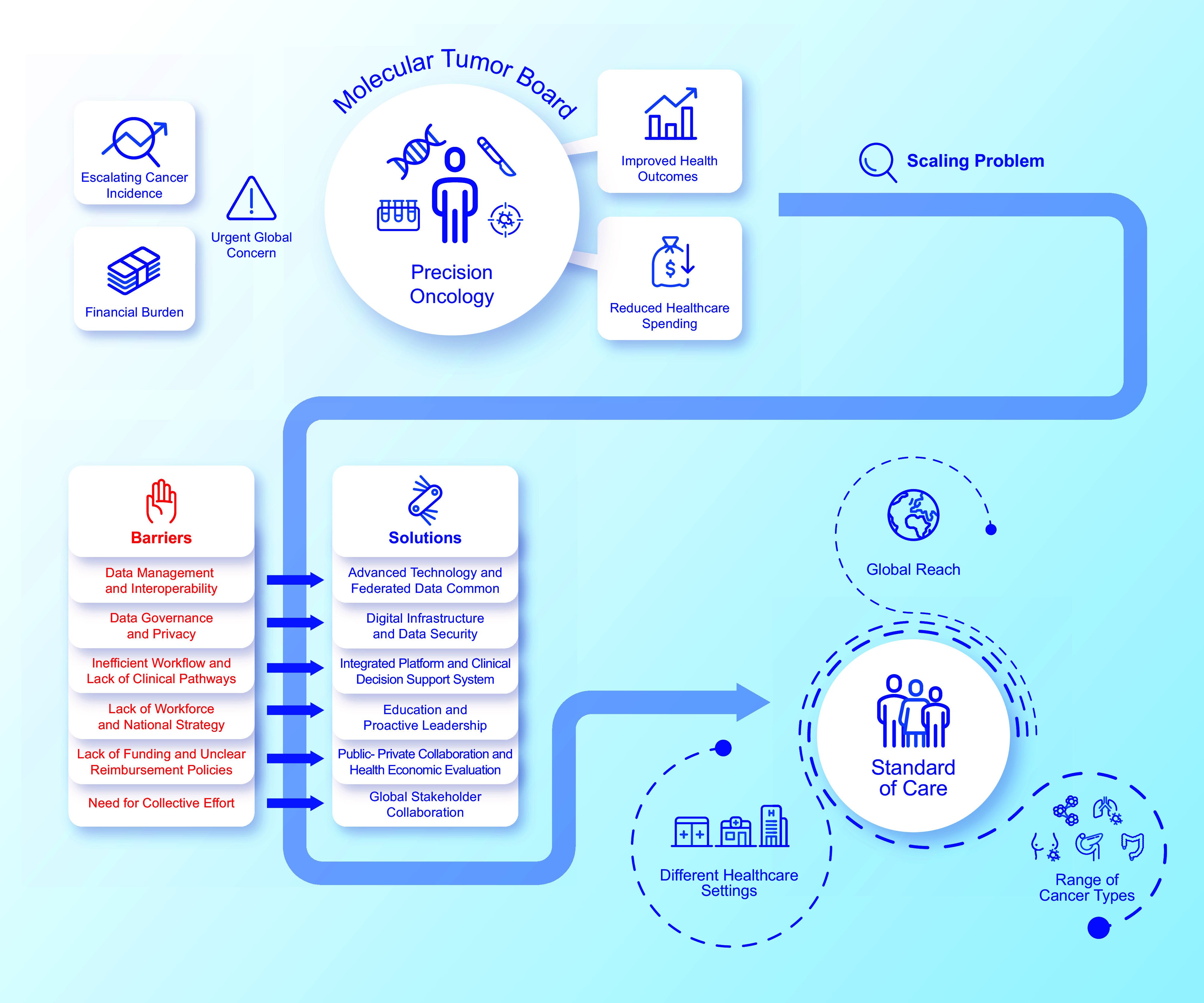

## Impact statement

As the global cancer prevalence and its associated financial costs escalate, cancer emerges as an urgent global concern necessitating cost-effective improvement in health outcomes that implicates a precision approach, giving the right treatment to the right patient at the right time and for the right duration. Despite the promise of precision oncology, progress in realising its transformative potential at scale remains slow due to the workforce, technical, clinical, regulatory and economic barriers. Here, we review the complexity of the scaling problem with comprehensive exploration of the different barriers and solutions that take forward a pragmatic and rational approach based on a minimum effective infrastructure to make precision oncology both scalable and cost-effective. These insights serve as a call for regional, national and global efforts, championing the integration of a broad range of fit-for-purpose health innovations into precision oncology practice in order to maximise the full potential of digital data-driven personalised approach for mainstream cancer care, regardless of regional or economic boundaries.

## Introduction

The scale of the oncology healthcare system in terms of the health economy is vast and expanding as cancer incidence and prevalence increase (Global Burden of Disease 2019 Cancer Collaboration, 2022). Cancer is a worldwide health burden at ‘pandemic scale’ that demands considerable resources for screening, diagnosis and multi-modality treatment. Concurrently, the economic burden of cancer care has become a pressing concern in many parts of the world (Chen et al., 2023). In the European Union (EU), cancer care costs are estimated to exceed €100 billion annually, reflecting the complexity and intensity of treatments required (European Commission, 2023a). Similarly, in the US, the National Institutes of Health reported that the overall cost of cancer was $183 billion in 2015 and is projected to increase to $246 billion by 2030, based only on population growth (Mariotto et al., 2020). In the Eastern Europe, Middle East and Africa regions, the cost varies significantly due to disparities in healthcare infrastructure and access to treatment, but the economic burden is equally substantial (Hofmarcher et al., 2023a). These numbers highlight the need for sustainable financing mechanisms to resource effective and efficient cancer treatment with equal access across these regions.

Advanced cancer genome-wide sequencing has discovered that each individual cancer evolves to become unique and complex, such that better outcomes are achieved when treatments are personalised to each individual cancer rather than delivered empirically (Walton et al., 2022; Hofmarcher et al., 2023b; Horgan et al., 2023; Tan et al., 2023). This level of functional complexity in cancer means that simple stratification at the standard of care level must be converted to the true and rare subtype personalisation requiring integrated solutions across regional and national populations. Moreover, cancer treatment resistance is common following treatment interventions, and this also requires multi-component strategies to circumvent, resulting in the emergence of multiple treatment lines for cancer care. Cancer care is therefore converging towards proactive, personalised care in a chronic disease pathway where cancer patients live well, over prolonged periods, with disease controlled by sequential interventions.

Unequal access to cancer care can fragment clinical outcomes and have potential societal impacts, which frequently increase health and economic disparities (Horgan et al., 2023; Lu et al., 2023; Richardson et al., 2023). First-world economies are already experiencing fragmentation of healthcare, and those who can afford access to new treatments are benefitting from the investment in drug development. With a specific biomarker-driven focus in drug development for selective mechanism-based drugs for each specific subset of a cancer, the vast industry investment required to develop a drug and test it in clinical trials carries considerable additional risks. Thus, any inadvertent pathway that limits drug access to an ever-decreasing patient cohort requires broader adoption across all economies to offset reduced returns. The high costs of drug development and the need for sufficient return on investment, especially for rarer variants, push the costs onto payers. To make the economics of drug development work for societies to reap the benefit of new transformational cancer treatments, there must be a proactive strategy for scaling up cost-effective access to new licenced treatments. This alignment is vital for patients needing specialised treatments and is not only for the pharmaceutical industry, as governmental regulators also impact on delivery and pricing. Therefore, scaling the collaboration and strategies for improving the economic viability of drug development are also essential for the delivery of the personalised medicine paradigm.

The drive for economies of scale in common cancers will also address the rarer cancer subsets of all cancers. This now presents an inflexion point that will impact on the extent and impetus for drug development for these rare targets. The challenge now faced by both public and private cancer care providers is how to deliver a precision oncology service that is the new ‘standard of care’, so that every patient with a cancer diagnosis can benefit from timely genomic testing and equal access to personalised therapy without polarising the health economy.

Failure to address the scaling problem for standard of care integration with precision oncology is a public health issue, with challenges that will be apparent to multidisciplinary teams (MDTs). These challenges involve labour-intensive MDT preparation, often requiring the collaboration of diverse hospital staff to compile data from various sources. Delivering the MDT tumour board outcome often faces hurdles such as inadequate discussion time, potentially compromising decision quality, and the influence of factors such as incomplete data, uneven participation and technical difficulties that hinder effective decision-making and coordination (Hammer et al., 2020, 2021). Existing innovative solutions provide avenues to solve some of these challenges, enabling system-wide efficiencies. By adopting technical support solutions tailored to MDT challenges, options emerge for utilising scaling solutions designed for decision-making, workforce coordination, and collaboration within MDT teams. In parallel, evidence about long-term value-for-money to ensure the sustainable implementation of personalised oncology as part of standard of care is necessary and expected for payers. A number of examples exist in developed and developing countries where attempts are being made to scale adoption of personalised oncology into routine care (Delnord et al., 2022; Liu et al., 2022; Vellekoop et al., 2022; Fasola et al., 2023; Stark et al., 2023).

## Scaling solutions to healthcare data requires pragmatic rationalisation

**Figure 1.**
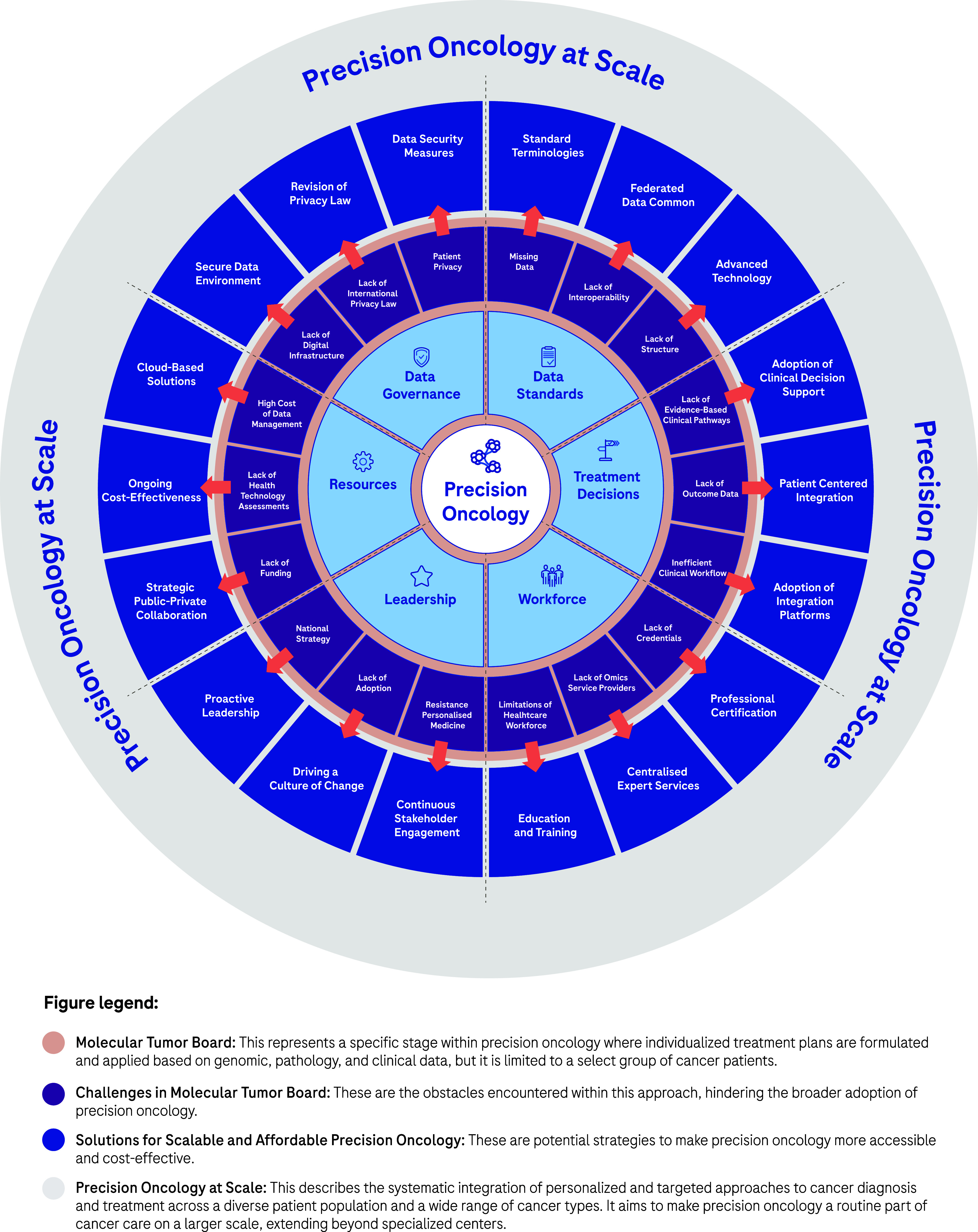
Precision Oncology at Scale.Integrated perspective addressing challenges and solutions to precision oncology at scale. See Figure legend.

An assumption often made is that the rapid advancement in data productivity can automatically support and inform individualised cancer care as the new standard (Williams et al., 2022). However, the reliance on disparate data sources generating heterogeneous data relevant to each patient’s cancer journey can often impede effective decision making, hamper coordination of care, increase variability in care and consequently lead to insufficient amounts of evidence to support reimbursement decisions (Liefaard et al., 2021; Baird et al., 2023). Structured digital innovations could address this issue by integrating the more relevant streams of healthcare data, aiming for specific improvements in the effectiveness of decision making. Importantly, efficient data exchange on a national and global scale while safeguarding patient privacy is also an imperative as sharing decisions and outcomes makes for effective widespread adoption of data-driven precision oncology. Although advancement in digital innovations holds the potential to accelerate the implementation of precision oncology at scale, their integration into routine clinical practice requires pragmatic rationalisation of a minimum specification and measured adoption in order to avoid ‘flooding’ the clinical data space and drowning the participants (Haynes et al., 2022; Patel et al., 2022, 2023). These pragmatic challenges are currently attributed to the diverse set of technical, governance, resource and leadership components that make up a standard of care MDT (Figure 1). Addressing these issues at scale is needed in order to provide further impetus for healthcare systems to capitalise on the full potential of their healthcare data and technological solutions for the benefit of patients and public health.

### Solutions to healthcare data challenges

Given the perceived benefits of control over access and security, healthcare organisations still rely on on-premise systems for data storage, integration and analysis. Yet, these on-premise systems are often inadequate for the massive and complex datasets currently used across precision oncology, expensive to scale and difficult to maintain (Dash et al., 2019). Another issue remains the quality of medical data with around 80% remaining in an unstructured format, buried in clinical notes and necessitating the utilisation of advanced techniques like natural language processing (NLP) at best, or even manual processing where such technologies are not sufficiently developed or available. Despite advances in data abstraction, there are concerns around the scalability of NLP in the standard of care setting that arise from its inability to perform cost-effectively. Moreover, dealing with missing and inconsistent data also remains a resource-intensive process that could result in disproportionate investment compared to the return in terms of clinical impact (Lawler et al., 2017; Kong, 2019; Savova et al., 2019). Streamlining how the data is structured and pre-processed needs to be considered for a cost-effective standard of care. For example, the minimal common oncology data elements (mCODE) endeavour is a good initiative that seeks to address the data standardisation challenge by providing a foundational data specification for oncology, employing standard and non-proprietary terminologies (Sweeney et al., 2023).

As individual healthcare systems continue to operate within these constraints, the lack of interoperability hinders the crucial aspect of data exchange (Lawler et al., 2017; Savova et al., 2019). The Global Alliance for Genomics and Health is a global stakeholder network that has developed a useful framework that aims to improve the interoperability of healthcare data, including genomics. Challenges to data sharing were mainly found to be financial, with lack of affordability being a substantial barrier to adoption. Despite this challenge, numerous country and region-specific initiatives have realised the potential of data sharing, including the need for avoiding a mandatory single data commons and repository, with many programmes adopting a more federated and pragmatic structure (Lawler et al., 2017).

Data analysis in precision oncology, like sequence alignment and variant discovery, are computationally demanding tasks. They do not need to be replicated in every cancer centre and can be organised with respect to economies of scale. The challenge, however, lies not only in detecting genetic alterations related to cancer progression, especially novel variants, but also in discerning their clinical relevance for tailored treatments. This involves interpreting these findings against a backdrop of the patient’s complete profile, including medical history, patient-reported outcomes, ethnicity, lifestyle, existing genomic data as well as documented effects of similar genomic variants from public databases (Schwartzberg, 2016; Sabapathy and Lane, 2018; Ryan et al., 2023). Despite this prerequisite based on the outcomes of MDT decisions, there is a noticeable scarcity of informed data when linking these treatment interventions to actual clinical and patient-centred outcomes.

### Solutions to data governance challenges

Ensuring the responsible storage, use and sharing of precision oncology data demands a robust data governance that concomitantly safeguards patient privacy and enables transparent data sharing necessary for clinical purposes and research, a ‘*sine qua non*’ for advancing precision oncology and improving patient outcomes (Balthazar et al., 2018). Nonetheless, it remains commonplace for patients to express concerns about potential misuse of their data (Sanderson et al., 2016), leading to potential exploitation and discrimination (Lowrance and Collins, 2007). In a survey conducted by Ghafur et al. (2020), it was observed that the more commercial the purpose of the receiving body, the more stakeholders varied in their willingness to share anonymised personal health information comparing the US and the UK. There remains no comprehensive international data privacy law, with de-identified data sets still containing patient-unique data that poses a risk for patient re-identification. Nonetheless, access control, data anonymization and cryptography were suggested as examples of combined systems and processes that can help prevent re-identification (Sweeney et al., 2023). Presently, there is a call for public standards that secure transparent data use and sharing and support patient understanding of how data is used and for what purposes. Increasingly, some experts are championing the concept of patient data ownership, enabled by blockchain technology, as a promising approach to navigate the complexities of data sharing and the vital task of safeguarding patients’ privacy (Montgomery, 2017). Such platforms give patients the ability to own, control, and decide by who, how and why their data will be used. Organisations like the Australian Genomics Health Alliance piloted platforms that allow patients to decide on their participation and use of their data for research. Although patient data ownership sounds promising, implementing it requires stronger health system infrastructure to successfully enable and support patient data ownership. This involves a digital infrastructure and determining whether either a centralised or decentralised data storage is more effective. It also requires the establishment of a patient identity ownership system, which could range from being limited to fully self-governed. Importantly, we cannot overlook the importance of patients’ knowledge and awareness about their own genomic data and the practice of clinical genomics and the necessity of comprehensive policies at both federal and state levels. The real-world adaptation to these barriers led to the re-evaluation of the EU’s General Data Protection Regulation (GDPR) with proposals for international secure data environments, clear responsibility delineations, and specific data analysis requirements (Bernier et al., 2023). To fully maximise the use of health data, NHS England recently invested £260 million in developing secure data environments (SDEs) as data storage and access platforms. Core aims are to overcome the data privacy and security issues when used for research and analysis, and to allow authorised users to access and analyse data without the raw data leaving the environment. Although increasing amounts of attention have been geared towards understanding and solving the data privacy issue, many questions remain unanswered on how enforced data privacy laws apply to accessing and exchanging sensitive data like genomics.

### Solutions to resource challenges

Given the rapid pace of technical development and the growing understanding of precision oncology, the incurred data costs are now less about the analysis pipelines but more about significant costs for storage, computation and backup of increasingly large amounts of sequencing and clinical data. In addition, the need for staff training on either updated or new IT functionalities places additional pressure on maintaining up-to-date operating systems with limited staff. Either a dedicated or a shared specialised team working across departments in one or more organisations is required to manage health IT infrastructure and enforce robust cybersecurity practices. Collaborations with digital experts and the use of centralised cloud-based solutions can both be helpful in ensuring healthcare organisation cybersecurity resilience (Abernethy et al., 2022; Sweeney et al., 2023). Failure to address these challenges may negatively impact patient safety and the quality of care delivery, leading to financial setbacks. After the WannaCry cyber attack on the UK National Health Service, Barts Health NHS Trust reported an estimate of £4.8 M required to offset the loss of income and the costs of hiring digital experts to support the recovery process post-attack. In the US, $6.2 billion is the annual cost for data beaches, as reported in a study by IBM and the Ponemon Institute (Ghafur et al., 2019). These figures underscore the importance of ongoing resource investment in IT budgets to ensure that current systems can be sustained securely and remain resilient.

Furthermore, for medical institutions to remain at the forefront of the ever-evolving precision oncology landscape, there is a pressing need for continuous innovation and the adoption of new solutions. The roadblocks here include limited funding and resources for research and development, institutional resistance to change, and regulatory challenges when introducing novel technologies. This highlights the need for potential strategic collaborations among academic institutions, industries, and healthcare providers in catalysing innovation and providing pragmatic and scalable solutions. Creating and nurturing a culture of continuous learning and innovation within these institutions is essential, but difficult to achieve despite public and private grant opportunities often spotlighted as key innovation drivers (Cesario et al., 2022).

### Solutions to the adoption challenges

Precision oncology is a disruptive innovation, necessitating a receptive context for change. Oftentimes, even when successfully implemented, the diffusion of innovation in healthcare organisations can be slow because of a disconnect between the objectives of health system administrators and frontline clinical teams. Administrative decisions, while well-intentioned, might not fully grasp the on-the-ground clinical complexities, leading to either potential misalignments in strategies or resistance to new clinical pathways. At the same time, frontline clinical teams may not have access to the context precipitating the need for certain administrative decisions. This disconnect underscores the importance of visionary leaders on both sides who can bridge these gaps by making precision medicine a priority and spearheading a clear vision and strategy. This necessitates a proactive leadership directed towards sharing clear goals and priorities where the individuals can see expected positive outcomes of the innovation. Furthermore, transitioning from one size fits all to a personalised approach demands from leaders to drive a culture of change, an attitude towards risk-taking and acceptance of failure, knowledge building and sharing. Berwick (2003) recommends that healthcare leaders who want to catalyse the rate of diffusion of innovations within their organisations should simplify the change process, find and support ‘innovators’, invest in ‘early adopters’, make early adopter activity observable and triable, trust and enable reinvention, create slack for change, and lead by example. However, the goal for scalability for precision oncology is achieving full adoption across the overwhelming majority of providers, and that will require a new cadre of leadership to deliver. The latter goal, however, requires substantial support to map the challenges and solutions at an individual provider level, utilising a common framework. In their roadmap for diffusion of innovation in healthcare, Balas and Chapman (2018) suggested a framework based on clinical practice guidelines, patient information, decision support, new incentives and supportive policies to facilitate widespread adoption of the innovation. More importantly, in order to achieve general access for standard of care, leaders should invest in the ‘laggards’ through pertinent accreditation requirements, public awareness campaigns, special quality improvement incentives, financial penalties and new liabilities (Balas and Chapman, 2018). Leaders should drive continuous stakeholder engagement from patients to policymakers and disseminate the evidence and lessons learned to ensure all perspectives are heard (Greenhalgh et al., 2004; Chanfreau-Coffinier et al., 2019; Shih et al., 2022). This would ultimately build credibility and drive compatibility of the innovation with the regulatory and access process (Malcarney et al., 2017).

## Scaling clinical implementation requires pragmatic rationalisation

Even though the clinical benefit of precision oncology has been realised in various cancers, its widespread implementation still faces numerous challenges (Dupont et al., 2021). The latter manifests at every stage of the patient’s journey and impacts multiple stakeholders along the way (Baird et al., 2023). The challenges include an already unprepared and overworked healthcare workforce with widespread disparities in education. Furthermore, the inefficiency of the clinical workflow makes aggregating and interpreting high-quality molecular and clinical data a challenging task. Moreover, the lack of evidence-based clinical pathways and restricted access to genomic testing and targeted therapies are broader systemic issues that add to this. Reimbursement and regulatory barriers exacerbate the situation with limited options for cost coverage, accessing off-label and experimental drugs, and uncertain impacts on healthcare utilisation. Lastly, low patient awareness, gaps in regional advocacy, reluctance among healthcare professionals and poor coordination among policymakers contribute to the depth of the scaling problem (Horgan, 2018; Chanfreau-Coffinier et al., 2019; Koleva-Kolarova et al., 2022; Baird et al., 2023; Fasola et al., 2023).

### Solutions to workforce challenges

One of the main challenges for precision oncology implementation in the clinical setting remains the readiness of healthcare professionals to adopt precision oncology guidelines (Christensen et al., 2016), with evidence suggesting this is greatly due to the lack of confidence in interpreting genetic information (Salari, 2009). An important step to tackle this hesitancy includes providing the necessary education and training to current and future professionals, starting at an undergraduate level. Medical schools should focus on reshaping their *curriculum* to offer courses that provide an understanding of the applications and benefits of precision medicine. Precision oncology is highly dependent on ever-increasing ‘-omics’ big data, which encompasses genomics, transcriptomics, proteomics and metabolomics data (Lamichhane and Agrawal, 2023). The analysis and interpretation of high-dimensional data require complex computational pipelines which depend on highly specialised professionals and experts in running and troubleshooting analysis. Key medical professionals in the MDT should at least become familiarised with the bioinformatics processing of these data and be knowledgeable in the conditions required for its interpretation.

Likewise, healthcare institutions should support training opportunities for all medical and operational stakeholders. For example, NHS England’s National Genomics Education resource has developed a Master’s programme in Genomic Medicine, with courses ranging from the basics of genetics to bioinformatics analysis. The programme offers an overview of the applications of precision medicine in disease areas such as cancer and rare disorders. Importantly, it also focuses on patient communication and engagement strategies, a core element of precision medicine (NHS England, 2014a). NHS England has also recently established the Genomics Training Academy (GTAC), which provides training and education to specialist genomics laboratories and clinical workforce, while simultaneously providing interprofessional learning between England and different countries (NHS England, 2014b).

Larger and collaboration-based initiatives are also needed to ensure continued cross-disciplinary education of healthcare professionals. These initiatives should foster exchange of new knowledge between different disciplines, such as genetics, bioinformatics, pharmacology, epidemiology and others (Martin-Sanchez et al., 2023). The International Consortium for Personalised Medicine (ICPerMed), for instance, is responsible for the organisation of precision medicine-related events and provides a platform to support communication on precision medicine, with the goal of promoting its funding, research and implementation (ICPerMed, 2017). Together, these educational initiatives strive to integrate precision medicine in clinical practice, thereby bridging the gap between evidence-based medicine and precision medicine. Certification and credentialing of providers, stakeholders and MDT participants are better supported by these broader initiatives, but this is only sustainable if the organisations delivering the courses strive to continuously validate their professional training and education offer.

### Solutions to scaling clinical support technologies

As discussed, addressing clinical workflow inefficiencies also necessitates a substantial investment in data integration platforms that import, integrate and aggregate various data types. On a practical level, clinicians would benefit from a comprehensive data view of the patient journey, streamlined care coordination and better decision-making with less administrative and cognitive burden (Noh et al., 2021; Patel et al., 2023). With the rising complexity of interpreting molecular and clinical data, there is an increasing demand for the adoption of clinical decision support systems (CDS) that can assist treatment choices, enhance adherence to clinical guidelines and reduce variability (Scudder et al., 2020; Serramito-Gómez et al., 2021; Halligan et al., 2023). Despite these technological developments, interpretations of complex precision oncology data still require the expertise of genomics specialists, which can be supported by current Molecular Tumour Boards (MTB). The intention here is that the molecular boards should be fully integrated with the ‘standard of care’ MDT. If local expertise is lacking, virtual MDT providers enabled by cloud-based platforms can overcome the time and space barriers, providing crucial expertise remotely for uncommon and advanced-stage tumours, streamline discussion and implementation of the MDT recommendations. This can standardise the approach of molecular testing and the interpretation across hospitals, reduce inconsistencies, reduce costs and improve the scalability of the interventions as a standard of care (Madhavan et al., 2018).

As precision oncology scales, enrolling patients to clinical trials should also become the standard of care. While only a few clinical trials may apply, matching patients’ genomic profiles to trial eligibility criteria remains a challenging task for oncologists because they must stay updated on the numerous active trials, which demands a substantial amount of time and resources. The difficulty in matching patients to clinical trials is mainly due to the basket design of precision oncology trials that enrols only patients with similar genomic changes but with different histologies, making recruitment across multiple departments remarkably complex. While MTBs have been useful for clinical trial enrolment, they cannot address the trial-matching challenge because they typically focus only on certain cancer types (Larson et al., 2021; Farhangfar et al., 2022). In an effort to solve this problem, academic cancer centres (Klein et al., 2022; Keller et al., 2023) and industries (Gardner et al., 2021; Haddad et al., 2021) invested in the development and implementation of a number of clinical trial matching solutions that can automate the process. However, the proprietary nature of these solutions limited their widespread adoption. Therefore, open-source trial matching solutions are perceived as valuable capabilities that can be leveraged cost-effectively by many institutions for patient enrolment in precision oncology trials (Klein et al., 2022).

While precision oncology uses individual genetic information to inform treatment decisions, patient-reported outcomes hold the promise to drive personalised patient-centred care and improve patients outcomes in both clinical trials (Basch et al., 2017) and real-world settings (Barbera et al., 2020a) while reducing healthcare utilisations (Barbera et al., 2020b). Despite these exciting outcomes, the integration of patient-reported outcomes (PROs) in oncology practice remains a challenge due to the stretched workforce and time constraints. In response to this operational challenge, there was an increasing use of electronic PROs (ePRO) apps for patients and providers that may or may not interface with a data warehouse to streamline the collection, analysis and interpretation of PROs. These digital tools enable clinicians to act immediately on reported symptoms when needed with discrete clinical intervention. Several bodies of evidence point out that electronic collection of PROs improves patient outcomes, including longer time on treatment, better quality of life, and higher probability of survival compounded with reduced healthcare service utilisation (Pritchett et al., 2023). In the US, the Centers for Medicare and Medicaid Innovation included ePRO in the Enhancing Oncology Model programme, suggesting that ePRO implementation should be the standard for high-quality cancer care (Basch et al., 2020). Despite these benefits, the adoption of ePROs remains limited essentially due to the faced workflow and technologic challenges. In response to this, the National Comprehensive Cancer Network Electronic Health Record and Oncology Advisory Group explored roll‐out readiness and provided 10 guiding principles that support healthcare organisations in designing data collection workflows, minimising the burden and maximising the action, mitigating disparities, monitoring and measuring outcomes and ensuring continuous engagements (Cracchiolo et al., 2023).

### Solutions for global stakeholders challenges

The journey ahead demands collective effort from researchers, providers, payers, policymakers and the public to propel broader access to precision oncology. National and international initiatives like Europe’s Beating Cancer Plan (European Commission, 2023a), EU Mission: Cancer (European Commission, 2023b), No One Missed, by the US LUNGevity Organisation (LUNGevity Foundation, 2023), the Lung Ambition Alliance and the US Cancer Moonshot (Singer, 2022) were formed to drive data sharing, scientific discovery, education and policy shaping (Baird et al., 2023). Another endeavour around data sharing is the creation of frameworks and standards for the responsible and secure sharing of genomic data led by the Global Alliance for Genomics and Health (GA4GH) (Bahcall, 2021).

To aid the standardisation of the reporting of clinically relevant genomic data, the European Society for Medical Oncology proposed the ESCAT scale in 2018. The implementation of this classification in clinical practice improved therapeutic tailoring for cancer patients in Molecular Tumour Board decisions (Martin-Romano et al., 2022). Furthermore, in order to strengthen the evidence base and validate the effectiveness of precision oncology interventions, the National Cancer Institute conducted, in collaboration with the ECOG-ACRIN Cancer Research Group, the Molecular Analysis for Therapy Choice (MATCH) trial, which is one of the largest tumour-agnostic, precision oncology trials undertaken to date. The MATCH trial, which evaluated the effectiveness of different targeted therapies based on the genetic changes found in patients’ tumours, met its signal-finding objective with 25.9% positivity (O’Dwyer et al., 2023) and led to the accelerated approval of the first and only BRAF/MEK inhibitor combination with a tumour-agnostic indication for solid tumours carrying the BRAF V600E mutation which drives tumour growth in more than 20 different tumour types (Turski et al., 2016).

### Solutions for health economic challenges

Among all the challenges discussed earlier, financing and reimbursement remain major barriers to the implementation of precision oncology at scale. Although public-private financing agreements (Polychronakos, 2012; Power et al., 2014) and performance-based reimbursement (De Souza et al., 2012) mechanisms were suggested as potential strategies (Koleva-Kolarova et al., 2022), the successful implementation of these strategies faced various issues. Among these, the disconnect between academic and private sectors (Gurwitz et al., 2009), the misalignment in research priorities nationally and internationally (Syme et al., 2015) as well as the ethical and legal issues surrounding data and sample sharing (Vis et al., 2017). Other issues revolve around generating outcome data and the inability to implement risk-sharing agreements across different healthcare systems, compounded with the health technology assessment frameworks, which are not fit to the uncertainty of precision oncology interventions (Regier et al., 2022). Koleva-Kolarova et al. (2023) formulated a comprehensive roadmap containing actionable recommendations for effective financing and reimbursement models for precision medicine across diverse healthcare systems within Europe. The key recommendations encompass boosting the public and philanthropic funding in research and development, establishing international research consortia and collaborative platforms for public-private research sharing, establishing a legal framework, creating extensive pan-European databases, utilising financial agreements and improving transparency in pricing and reimbursement. These recommendations are intended to guide health authorities in developing a strategic sequence of policy measures that democratise access to cost-effective precision medicine standards of care.

## Future directions in precision oncology

The path to the successful implementation of precision oncology at scale demands integrated efforts and strategies. An imperative in this journey is the need for leadership to coordinate the integrated efforts of technologists, clinicians, scientists, hospital administrators, payers and policymakers. As such, solutions to the scaling problem now point to combining the skills, capabilities and experience needed to implement precision oncology and address barriers that hinder the wider adoption and polarise the cost. It is also vital for the healthcare system to focus investment into basic, necessary and pragmatic digital infrastructure, and to manage sustainable resources so that the healthcare provider organisations are able to adopt, integrate and manage advanced health IT systems. Beyond basic technology, refining clinical leadership, education and workflows is essential for enabling healthcare professionals to deliver effective personalised services that improve outcomes and maximise return on investment for both healthcare systems and society. Continuous up-skilling of the clinical workforce is vital for proficient advanced digital health innovation and understanding of the nuances of precision oncology. To achieve the scale required for cancer, bridging the gap between private and public providers and payers should further enhance mutual awareness and trust. Macroeconomic sustainability of these efforts is critical, which necessitates an adaptation of the regulatory framework and establishment of resilient funding mechanisms, coupled with comprehensive health economic evaluations that assess the cost-effectiveness of precision oncology interventions. Addressing these key areas has the potential to realise the transformative potential of precision oncology at scale and in an affordable way as the ‘standard of care’ for cancer.

## Data Availability

Data availability is not applicable to this article as no new data were created or analysed in this study.
