# Supplementary material for: Challenges and solutions to system-wide use of precision oncology as the standard of care paradigm
Source: Camb Prism Precis Med. 2024 Mar 26;2:e4. doi: 10.1017/pcm.2024.1 (PMC11062796; doi:10.1017/pcm.2024.1)
Supplement: Peer review history

# Editor report: Recommendation: Challenges and solutions to system-wide use of precision oncology as the standard of care paradigm — R0/PR2

**DOI:** 10.1017/pcm.2024.1.pr2

**Affiliations:** Clinical Ethics, Law and Society, Wellcome Trust Centre for Human Genetics, United Kingdom of Great Britain and Northern Ireland

No accompanying comment.

# Editor report: Decision: Challenges and solutions to system-wide use of precision oncology as the standard of care paradigm — R0/PR3

**DOI:** 10.1017/pcm.2024.1.pr3

**Affiliations:** Institute of Cardiovascular & Medical Sciences, University of Glasgow College of Medical Veterinary and Life Sciences, United Kingdom of Great Britain and Northern Ireland

No accompanying comment.

# Editor report: Recommendation: Challenges and solutions to system-wide use of precision oncology as the standard of care paradigm — R1/PR5

**DOI:** 10.1017/pcm.2024.1.pr5

**Affiliations:** Clinical Ethics, Law and Society, Wellcome Trust Centre for Human Genetics, United Kingdom of Great Britain and Northern Ireland

No accompanying comment.

# Editor report: Decision: Challenges and solutions to system-wide use of precision oncology as the standard of care paradigm — R1/PR6

**DOI:** 10.1017/pcm.2024.1.pr6

**Affiliations:** Institute of Cardiovascular & Medical Sciences, University of Glasgow College of Medical Veterinary and Life Sciences, United Kingdom of Great Britain and Northern Ireland

No accompanying comment.
